# Mesenchymal Stromal Cells From Emphysematous Donors and Their Extracellular Vesicles Are Unable to Reverse Cardiorespiratory Dysfunction in Experimental Severe Emphysema

**DOI:** 10.3389/fcell.2021.661385

**Published:** 2021-05-31

**Authors:** Mariana A. Antunes, Cassia L. Braga, Tainá B. Oliveira, Jamil Z. Kitoko, Ligia L. Castro, Debora G. Xisto, Mariana S. Coelho, Nazareth Rocha, Rodrigo P. Silva-Aguiar, Celso Caruso-Neves, Eduarda G. Martins, Clara Fernandes Carvalho, Antônio Galina, Daniel J. Weiss, José R. Lapa e Silva, Miquéias Lopes-Pacheco, Fernanda F. Cruz, Patricia R. M. Rocco

**Affiliations:** ^1^Laboratory of Pulmonary Investigation, Carlos Chagas Filho Institute of Biophysics, Federal University of Rio de Janeiro, Rio de Janeiro, Brazil; ^2^National Institute of Science and Technology for Regenerative Medicine, Rio de Janeiro, Brazil; ^3^Laboratory of Inflammation and Immunity, Paulo Goes Institute of Microbiology, Federal University of Rio de Janeiro, Rio de Janeiro, Brazil; ^4^Department of Physiology and Pharmacology, Fluminense Federal University, Niterói, Brazil; ^5^Laboratory of Biochemistry and Cell Signaling, Carlos Chagas Filho Institute of Biophysics, Federal University of Rio de Janeiro, Rio de Janeiro, Brazil; ^6^Leopoldo De Meis Institute of Medical Biochemistry, Federal University of Rio de Janeiro, Rio de Janeiro, Brazil; ^7^Department of Medicine, College of Medicine, University of Vermont, Burlington, VT, United States; ^8^Institute of Thoracic Medicine, Clementino Fraga Filho University Hospital, Federal University of Rio de Janeiro, Rio de Janeiro, Brazil

**Keywords:** cell therapy, COPD, mesenchymal stromal cells, extracellular vesicles, inflammation, macrophages, mitochondria

## Abstract

Although bone marrow-derived mesenchymal stromal cells (BM-MSCs) from patients with chronic obstructive pulmonary disease (COPD) appear to be phenotypically and functionally similar to BM-MSCs from healthy sources *in vitro*, the impact of COPD on MSC metabolism and mitochondrial function has not been evaluated. In this study, we aimed to comparatively characterize MSCs from healthy and emphysematous donors (H-MSCs and E-MSCs) *in vitro* and to assess the therapeutic potential of these MSCs and their extracellular vesicles (H-EVs and E-EVs) in an *in vivo* model of severe emphysema. For this purpose, C57BL/6 mice received intratracheal porcine pancreatic elastase once weekly for 4 weeks to induce emphysema; control animals received saline under the same protocol. Twenty-four hours after the last instillation, animals received saline, H-MSCs, E-MSCs, H-EVs, or E-EVs intravenously. *In vitro* characterization demonstrated that E-MSCs present downregulation of anti-inflammatory (TSG-6, VEGF, TGF-β, and HGF) and anti-oxidant (CAT, SOD, Nrf2, and GSH) genes, and their EVs had larger median diameter and lower average concentration. Compared with H-MSC, E-MSC mitochondria also exhibited a higher respiration rate, were morphologically elongated, expressed less dynamin-related protein-1, and produced more superoxide. When co-cultured with alveolar macrophages, both H-MSCs and E-MSCs induced an increase in iNOS and arginase-1 levels, but only H-MSCs and their EVs were able to enhance IL-10 levels. *In vivo*, emphysematous mice treated with E-MSCs or E-EVs demonstrated no amelioration in cardiorespiratory dysfunction. On the other hand, H-EVs, but not H-MSCs, were able to reduce the neutrophil count, the mean linear intercept, and IL-1β and TGF-β levels in lung tissue, as well as reduce pulmonary arterial hypertension and increase the right ventricular area in a murine model of elastase-induced severe emphysema. In conclusion, E-MSCs and E-EVs were unable to reverse cardiorespiratory dysfunction, whereas H-EVs administration was associated with a reduction in cardiovascular and respiratory damage in experimental severe emphysema.

## Introduction

Chronic obstructive pulmonary disease (COPD) is among the three leading causes of death worldwide, claiming over 3 million lives annually (Global Initiative for Chronic Obstructive Lung Diseases [GOLD], 2020). It is characterized by persistent respiratory symptoms and irreversible airflow limitations caused by meaningful exposure to noxious gases or particles that lead to small airway disease (chronic bronchitis) and/or extensive disruption of the alveolar walls with enlargement of airspaces (emphysema) (Barnes et al., 2015; Global Initiative for Chronic Obstructive Lung Diseases [GOLD], 2020). In emphysema, proteolysis of elastin and other extracellular matrix (ECM) components leads to loss of elastic recoil and promotes chemoattractant activity, which perpetuates inflammatory responses and ultimately results in extensive tissue remodeling (Kulkarni et al., 2016). With disease progression, individuals with COPD may also develop extrapulmonary abnormalities, such as pulmonary arterial hypertension and remodeling of the right ventricular structure, which have a negative impact on their quality of life and survival. Despite a better understanding of the pathological mechanisms in COPD, there is still no effective therapy to prevent or reverse progressive cardiopulmonary dysfunction in affected individuals (Barnes et al., 2015).

Mesenchymal stromal cell (MSC) therapy has demonstrated immunomodulatory and regenerative actions in experimental models of lung diseases by secreting soluble trophic factors and extracellular vesicles (EVs) and by transferring their mitochondria via tunneling nanotubes (Islam et al., 2012; Guan et al., 2013; Antunes et al., 2014; Jackson et al., 2016; Morrison et al., 2017; Poggio et al., 2018; Lopes-Pacheco et al., 2020). Both EVs and mitochondrial transfer were able to induce protective and antimicrobial actions, recover alveolar bioenergetics, and enhance macrophage phagocytic capacity in *in vitro* and *in vivo* models of acute respiratory distress syndrome (Islam et al., 2012; Jackson et al., 2016; Morrison et al., 2017). In experimental emphysema induced by cigarette smoke, bone marrow-derived MSCs (BM-MSCs), which can be chemoattracted to sites of injury by inflammatory chemokines and by matrikines—e.g., collagen present in the ECM that can induce higher adhesion, survival and proliferation of MSCs (Somaiah et al., 2015), as well as elastin fragments that promote higher MSC adhesion (Jin et al., 2016), reduced cell apoptosis counts and levels of pro-inflammatory mediators and proteases in lung tissue (Guan et al., 2013). Evidence suggests that biophysical features involved in MSC culture may also impact subsequent immunomodulation by the MSCs (Wong et al., 2020). We have previously demonstrated that systemic administration of BM-MSCs reduced lung inflammation and damage and induced macrophage polarization to an anti-inflammatory M2 profile in moderate experimental emphysema induced by elastase (Antunes et al., 2014). Furthermore, two doses of BM-MSCs (1-week apart) administered intratracheally were able to partially reverse cardiorespiratory dysfunction in experimental emphysema (Poggio et al., 2018). Such promising effects observed in experimental settings have prompted clinical investigations. However, despite demonstrating safety, efficacy was not clearly demonstrated in individuals with moderate to severe COPD in early stage clinical trials (Weiss et al., 2013; Stolk et al., 2016), suggesting that MSC-based approaches still need to be better understood and optimized to achieve success in clinical practice.

A functional characterization of BM-MSCs from individuals with COPD and non-COPD controls demonstrated no significant differences in terms of morphology, migration, and proliferation, except an increased potential for adipocyte differentiation in the former (Broekman et al., 2016). Both groups also exhibited similar levels of inflammatory mediators and growth factors, which indicates that these cells might be used autologously for the treatment of COPD (Broekman et al., 2016). However, the potential effects of MSCs from COPD donors should be further characterized, in particular in relation to their EVs and mitochondrial function, because these have been considered key mechanisms by which these cells exert their therapeutic actions.

The present study aimed to compare the therapeutic potential of MSCs and EVs from healthy (H) and emphysematous (E) donors in experimental emphysema. H-MSCs and E-MSCs were characterized with regard to EV morphology, mitochondrial function, and expression levels of selected biomarkers. In parallel, the ability of MSCs and their EVs to promote mitochondrial transfer and alveolar macrophage polarization was evaluated *in vitro*. Finally, MSCs or EVs were administered intravenously in a murine model of elastase-induced severe emphysema to assess their therapeutic effects on lung function, histology, inflammatory biomarkers, and cardiac function.

## Materials and Methods

This study was approved by the Ethics Committee of the Federal University of Rio de Janeiro Health Sciences Center (CEUA-UFRJ: 014/14, Rio de Janeiro, Brazil). All animals received humane care by trained veterinarians and veterinary staff in compliance with the “Principles of Laboratory Animal Care” formulated by the National Society for Medical Research and the U.S. National Academy of Sciences *Guide for the Care and Use of Laboratory Animals*.

### Study Design

Seventy-eight female C57BL/6 mice (weight 20–25 g, age 2–3 months) were used. BM-MSCs and EVs were obtained from 8 animals [*n* = 4/type: healthy (H) or emphysematous (E)], and 10 animals were used to obtained alveolar macrophages (AMs) from bronchoalveolar lavage fluid (BALF). The 60 remaining animals were used for evaluation of cardiovascular function, lung mechanics and histology, cytokine levels, and neutrophil content in lung tissue. Mice were randomly assigned into two main groups: control (C) and emphysema (ELA). Mice in the ELA group received pancreatic porcine elastase (PPE; 0.2 IU in 50 μL of saline, intratracheally) once a week for 4 consecutive weeks; the C group received saline (50 μL) using the same protocol (Supplementary Figure 1). Tracheal instillations during the development of this emphysema model did not result in local inflammation or increased mortality rate.

### MSC Isolation and Characterization

One week after the last instillation of saline or PPE, C and ELA mice were anesthetized with intravenous ketamine (25 mg/kg) and xylazine (2 mg/kg). Bone marrow cells were isolated from femurs and tibias and then pooled. Cells were cultured (T25 culture flasks; TPP, Schaffhausen, Switzerland) with Iscove’s modification of Dulbecco’s medium (IMDM; GE Healthcare Life Sciences, Logan, UT, United States) supplemented with 10% fetal bovine serum (FBS) (GE Healthcare Life Sciences), 10% horse serum (GE Healthcare Life Sciences), 1% penicillin/streptomycin (Thermo Fisher Scientific, Waltham, MA, United States), and 2 mM L-glutamine (Thermo Fisher Scientific) in a humidified incubator (5% CO_2_, 37°C). On day 3, the medium was changed and non-adherent cells were removed. Adherent cells proliferated and upon reaching 80% confluence, they were passaged with 0.05% trypsin-EDTA solution (Gibco, Thermo Fisher Scientific) and then maintained in supplemented IMDM. MSCs were used at passages 4–6. At the fourth passage, approximately 1 × 10^6^ cells were characterized as MSCs according to the International Society of Cellular Therapy Consensus.

### EV Extraction and Characterization

In order to collect EVs released from H-MSCs and E-MSCs, cells were cultured with serum-free medium for 48 h. The medium was collected and centrifuged at 2,000 × *g* for 20 min at 4°C to remove cellular debris, followed by 2 rounds of ultracentrifugation (100,000 × *g*) for 1 h each at 4°C. The precipitate was collected and suspended in saline, except for the NanoSight analysis, for which EVs were suspended in filtered phosphate-buffered saline (PBS). The total protein content of the EV fraction was quantified by Bradford’s assay. Analysis of the absolute size distribution and concentration of EVs was performed using NanoSight NS300 (Malvern Instruments Ltd, Malvern, United Kingdom). The analysis settings were optimized using filtered PBS as control and kept constant between samples. The measurement conditions for nanoparticle tracking analysis (NTA) were as follows: three measurements per sample (30 s/measurement), temperature 25°C, viscosity 0.9 cP, 25 frames per second. Each video was analyzed to give the mean, mode, median, and estimated concentration for each particle size. The samples were diluted to obtain the appropriate number of particles (proportional to 1 × 10^6^ MSCs/500 μL), which was proportional to the number of MSCs administered to the animals in the *in vivo* experiments.

Scanning electron microscopy of BM-MSCs was performed to evaluate the presence of EVs released from MSC surfaces. After collection of conditioned media for EV isolation, H-MSCs and E-MSCs were fixed in 2.5% glutaraldehyde in 0.1 M sodium cacodylate buffer (pH 7.2) for 2 h and washed twice with cacodylate buffer. After fixation with OsO_4_ and FeCNK solution (1:1) for 45 min, MSCs were then dehydrated in an ethanol series, critical-point-dried in CO_2_, mounted on stubs, sputter-coated with a thin gold layer, and observed under a JEOL JSM5310 electron microscope (JEOL, Peabody, MA, United States).

For further characterization, flow cytometry was performed with the addition of aldehyde/sulfate latex beads (Thermo Fisher Scientific, United Kingdom). The latex beads were used to provide a reference point for comparison with the EVs in FSC vs. SSC plots. A total of 50 μL of each isolated fraction were incubated with 0.25 μL of aldehyde/sulfate-latex beads (ø = 4μm; 5.5 × 10^6^ particles/ml) for 20 min at room temperature. Thereafter, 1 mL of FACS buffer [PBS supplemented with 0.1% BSA (Roche) and 0.01% NaN_3_ (G-Biosciences)] was added and the sample was incubated overnight on rotation. Bead-coupled EVs were centrifugated at 20,000 × *g* for 10 min, washed with 1 mL of FACs buffer, and centrifuged again. The pellet was then resuspended with 100 μL of FACS buffer and stained with anti-CD63 (Biolegend) PE-conjugated or anti-CD81 (Biolegend) PE-conjugated primary antibodies for 45 min at 4°C. After incubation, H-EVs and E-EVs were washed twice and resuspended in FACS buffer. Gating of EV-decorated beads 4 μm in diameter was performed based on FCS/SSC parameters, so that unbound EVs or possible antibody aggregates were excluded from the analysis. Data acquistion was performed in a FACS CantoII flow cytometer using FACSDiva software, and data analysis was performed using FlowJo X 10.0.7 software (Tree Star, Ashland, OR, United States).

### Measurement of MSC Mitochondrial Respiration

H-MSCs and E-MSCs (1 × 10^6^ cells per sample) were dissociated in 2 mL of plain IMDM (at 37°C) in the chamber of a high-resolution respirometer (Oroboros Oxygraph O_2_k, Oroboros Instruments, Innsbruck, Austria). Measurement of O_2_ consumption (expressed in pmol O_2_/s/10^6^ cells) was carried out under basal conditions after addition of oligomycin (2 μg/mL; Sigma-Aldrich, St. Louis, MO, United States) to inhibit F_0_F_1_-ATPsynthase, or after successive additions of 1 μM carbonyl cyanide 4-(trifluoromethoxy)phenylhydrazone (FCCP; Sigma-Aldrich) to uncouple mitochondrial respiration.

### MitoSOX-Based Flow Cytometry

A MitoSOX red (Thermo Fisher Scientific)-based flow cytometric assay was used to detect mitochondrial reactive oxygen species (ROS) in H-MSCs and E-MSCs, as described elsewhere (Kauffman et al., 2016). Samples were evaluated on a FACSCalibur flow cytometer (BD Biosciences Immunocytometry Systems, San Jose, CA, United States). The mean fluorescence intensity and percentage of stained cells were determined using FlowJo X 10.0.7 software (Tree Star, Ashland, OR, United States).

### Transmission Electron Microscopy

After 48 h of culture in serum-free medium, H-MSCs and E-MSCs were collected by centrifugation, washed in PBS, and fixed for 24 h at 4°C in 2.5% glutaraldehyde in 0.1 M cacodylate buffer. After fixation, MSCs were washed with cacodylate buffer and post-fixed in a solution containing 1% OsO_4_, 1.25% potassium ferrocyanide, 5 mM CaCl_2_, and 0.1 M cacodylate buffer for 30 min. MSCs were then washed in the same buffer. Ultrathin sections were stained with uranyl acetate and lead citrate and observed under a Zeiss 900 electron microscope (Carl Zeiss, Jena, Germany).

### Quantification of mRNA Expression Levels of Biomarkers

Quantitative real-time reverse transcription polymerase chain reaction (qRT-PCR) was performed to assess the mRNA levels of several biomarkers as described previously (Abreu et al., 2019). Briefly, total RNA of MSCs was extracted (RNeasy Plus Mini Kit, QIAGEN, Hilden, Germany) and the RNA concentration measured by spectrophotometry (Nanodrop ND-1000 system; Thermo Scientific). cDNA synthesis was performed with a High-Capacity cDNA Reverse Transcription Kit (Thermo Fisher Scientific) according to the manufacturer’s instructions. Relative mRNA levels were measured with the BRYT green system (Promega, Madison, WI, United States) in a PCR Mastercycler ep Realplex (Eppendorf, Hamburg, Germany). The relative expression of each gene was calculated as a ratio of the gene under study to the control gene *36B4* and expressed as fold change relative to the C group (2^–ΔΔ*Ct*^). The mRNA expression of the following genes was analyzed: HGF, IDO-1, IL-1RN, IL10, TGF-β, TSG-6, VEGF, CAT, SOD, Nrf2, GSH, MFN1, MFN2, and DNM1. The list of primers is described in Supplementary Table 1.

### Immunoblotting

H-MSCs and E-MSCs at passages 4–6 were cultured in 6-well plates for 3 days. Cells were then resuspended in lysis buffer containing 20 mm HEPES, 2 mm EGTA, 1% Triton X-100, phosphatase inhibitors (5 mM Na_4_P_2_O_7_, 50 mM NaF, 5 mM Na_3_VO_4_, 10 mM sodium β-glycerophosphate) and a protease inhibitor cocktail (Sigma-Aldrich). After 30 min, cells were cleared by centrifugation at 15,000 × *g* for 10 min at 4°C. Proteins was resolved in 9% SDS-PAGE gel and transferred to polyvinylidene difluoride membranes (Millipore). Detection of MFN1 (#ab57602, Abcam), MFN2 (#ab56889, Abcam), and DRP-1 (#ab156951, Abcam) were performed according to manufacturer instructions. Beta-actin (#ab8227, Abcam) was used as a loading control. Secondary antibody was anti-rabbit IgG (#7074, Cell Signaling Technology). Detection was performed with ECL Prime (Cytiva). Images were acquired using ImageQuant LAS 4000 (Cytiva) and analyzed in ImageJ software.

### Co-culture of MSCs or EVs With Alveolar Macrophages

AMs were obtained from the BALF of 10 healthy C57BL/6 mice as previously described (Abreu et al., 2019). Briefly, BALF was centrifuged at 300 × *g* for 10 min, and the cellular pellet was washed with PBS. Cells were re-suspended in red blood cell lysis buffer (8.3 g NH_4_Cl, 1 g KHCO_3_, 1.8 mL 5% EDTA in 1,000 mL distilled water) for 5 min at room temperature and centrifuged again at 300 × *g* for 10 min. The pelleted cells were re-suspended and cultured in a 12-well culture plate at 37°C with 5% CO_2_ at a concentration of 10^5^ cells per well in 1 mL of RPMI 1640 medium (Sigma, St. Louis, MO, United States) supplemented with 10% FBS, 1 mM pyruvate, 1% non-essential amino acids, 14 mM glucose, 17.9 mM NaHCO_3_, 10 mM HEPES, and penicillin/streptomycin. After 2 h of incubation, non-adherent cells were washed off with PBS, and the medium was replaced. During the experiments, MSCs were counted in a Neubauer chamber and plated in 6-well plates at 5 × 10^4^ per well. MSC-derived EVs were added to the culture plate as a proportional dose obtained from 5 × 10^4^ MSCs per well. AMs were also counted and plated at 1 × 10^6^ per well (1:20 ratio). After 24 h, AMs were processed for quantification of mitochondrial transfer from MSCs by flow cytometry or for molecular biology analysis.

To evaluate mitochondrial transfer, MSCs were labeled by incubating the cells with a MitoTracker Deep Red FM probe (APC) (Thermo Fisher Scientific, catalog no. M22426) for 30 min at 37°C in 5% CO_2_ as previously described (Jackson and Krasnodembskaya, 2017). The staining solution was then removed, and MSCs were washed twice with PBS. EVs obtained from previously stained MSCs were also extracted (following the same protocol) for co-culture experiments. Stained MSCs or EVs were co-cultured with AMs for 24 h. Thereafter, AMs were washed twice with PBS, blocked with anti-CD16/32, and stained with monoclonal anti-mouse CD45 FITC antibody (eBiosciences, San Diego, CA, United States). Non-stained MSCs or EVs were used as native controls. All data were acquired in a FACSCalibur flow cytometer (BD Biosciences Immunocytometry Systems) and analyzed using FlowJo X 10.0.7 software (Tree Star).

To evaluate the ability of MSCs or EVs to promote AM polarization, cells were co-cultured from 24 h. Thereafter, cells were washed twice with cold PBS followed by gentle detachment from the plastic surface using a cell scraper. Cells were then incubated with 2% anti-CD11b antibody in PBS for 20 min. Cell suspensions were washed with PBS, pelleted by centrifugation (600 × *g* for 5 min), re-suspended with Dynabeads solution (Thermo Fisher Scientific) in PBS and, after a 20-min incubation, transferred to a magnetic particle concentrator. Isolated AMs were washed three times with PBS, pelleted by centrifugation, and processed for qRT-PCR analysis. The mRNA expression of arginase-1, inducible nitric oxide synthase (iNOS), matrix metalloprotease-9 (MMP9), and IL-10 was assessed (Supplementary Table 1).

To assess the potential of EVs to modify AM bioenergetics by mitochondrial transfer, they were co-cultured from 24 h (ratio of EVs proportional to 1 MSC to each 20 AMs plated in the well) in 6-well plates. Thereafter, AMs were washed with cold EDTA 2 mM in PBS for gentle cell detachment from the plastic surface. AM suspensions were pelleted by centrifugation (600 × *g* for 5 min), and dissociated with 2 mL of plain RPMI 1640 medium (at 37°C) in the chamber of a high-resolution respirometer (Oroboros Oxygraph O_2_k, Oroboros Instruments, Innsbruck, Austria). Measurement of O_2_ consumption was carried out as previously described in item Measurement of MSC Mitochondrial Respiration.

### Animal Preparation and Induction of Emphysema for *in vivo* Experiments

Twenty-four hours after the last instillation of elastase, when features of emphysema were evident (Oliveira et al., 2016), animals were randomized to receive systemic administration of either saline solution (0.9% NaCl, 50 μL, SAL), H-MSCs or E-MSCs (1 × 10^6^ cells in 50 μL saline), EVs derived from H-MSCs (H-EV), or E-MSCs (E-EV); EVs obtained from 1 × 10^6^ cells were suspended in 50 μL of saline per animal. Mice were anesthetized with sevoflurane, the left jugular vein of each mouse was exposed by ventral neck dissection, and MSCs or EVs were slowly injected.

### Echocardiography

For echocardiographic assessment of cardiac function, mice were anesthetized with 1.5% isoflurane, shaved over the precordial region, and examined with a Vevo 770 apparatus (VisualSonics, Toronto, ON, Canada) coupled to a 30 MHz transducer. Images were obtained from the parasternal view. M-mode images showed left ventricular muscle thickness. One long-axis and four short-axis B-dimensional views of both ventricles were acquired to calculate the right ventricular area. Pulsed-wave Doppler was used to measure the pulmonary artery acceleration time (PAT) and pulmonary artery ejection time (PET). All procedures followed American Society of Echocardiography and European Association of Cardiovascular Imaging recommendations (Lang et al., 2005; Oliveira et al., 2016).

### Lung Mechanics

Seven days after administration of saline, MSCs, or EVs, mice were sedated with diazepam (5 mg/kg, intraperitoneally), anesthetized with sodium thiopental (20 mg/kg intraperitoneally), tracheotomized, paralyzed with vecuronium bromide (0.005 mg/kg, intravenously), and ventilated with a constant-flow ventilator (Samay VR15, Montevideo, Uruguay). The anterior chest wall was surgically removed, and a positive end-expiratory pressure of 2 cmH_2_O was applied. Airflow and transpulmonary pressure were measured, and lung mechanics were analyzed by the end-inflation occlusion method. After end-inspiratory occlusion, plateau pressure (Pplat) corresponded to the elastic recoil pressure of the lung. Static lung elastance (Est,L) was determined by dividing Pplat by the tidal volume (Bates et al., 1988; Antunes et al., 2014; Poggio et al., 2018). All data were analyzed in ANADAT software (RHT-InfoData, Montreal, QC, Canada) and are expressed as means ± SD of 7–10 mice per group.

### Lung Morphometry

At the end of the experiment, laparotomy was performed, and heparin (1,000 IU) was injected into the vena cava. The trachea was clamped at end-expiration, and the abdominal aorta and vena cava were transected, producing terminal bleeding for euthanasia. The right lung was then removed, fixed in 10% buffered formalin, and embedded in paraffin. Sections (4-μm thick) were stained with hematoxylin-eosin. Airspace enlargement was assessed by measuring the mean linear intercept (Lm) between alveolar walls at a magnification of × 400 (Hsia et al., 2010; Padilha et al., 2015; Henriques et al., 2016). The percentage of neutrophils in pulmonary tissue was determined by the point-counting technique across 10–20 random, non-coincident microscopic fields (Hsia et al., 2010; Padilha et al., 2015; Henriques et al., 2016). Collagen fibers in airway and walls of blood vessels in interlobular septa (Masson’s trichrome stain), as well as elastic fibers in alveolar septa (Weigert’s resorcin fuchsin method with oxidation), were computed using ImagePro Plus 6.0 software (Antunes et al., 2014; Poggio et al., 2018).

### Enzyme-Linked Immunosorbent Assay (ELISA)

Lung-tissue homogenates were used for cytokine quantification. Sandwich ELISAs for IL-10 and IL-1β (Peprotech, Rocky Hill, NJ, United States) were performed as per the manufacturer’s instructions, as was an assay for TGF-β (BioLegend, San Diego, CA, United States). Results were normalized by the total amount of protein assessed by the Bradford method in each sample and are expressed as picograms/milligram.

### Statistical Analysis

Sample size was based on pilot studies and on our experience with models of elastase-induced emphysema (Antunes et al., 2014; Oliveira et al., 2016; Poggio et al., 2018). Data were tested for normality using the Kolmogorov–Smirnov test with Lilliefors’ correction, and the Levene median test was used to evaluate homogeneity of variances. If both conditions were satisfied, differences between groups at *in vivo* analysis were assessed using one-way ANOVA followed by Bonferroni’s test. For non-parametric results, the Kruskal–Wallis test followed by Dunn’s test was used. For results of *in vitro* analysis, the Student *t*-test and Mann–Whitney *U* test were used as appropriate. *In vitro* experiments were performed using three or four different samples in each condition (H or E, MSC or EV). In both *in vivo* and *in vitro* analyses, parametric data were expressed as means ± standard deviation, and non-parametric data as medians (interquartile range). All tests were carried out in GraphPad Prism version 6.07 (GraphPad Software, San Diego, CA, United States). Significance was established at *P* < 0.05.

## Results

### Characterization of MSCs and Their EVs Obtained From Healthy and Emphysematous Donors

In accordance with International Society for Cellular Therapy guidelines, H-MSCs and E-MSCs exhibited characteristic spindle-shaped morphology, adherence to plastic culture flasks (data not shown), capacity to differentiate into osteoblasts (Supplementary Figure 2), and expression of MSC-specific cell-surface markers (similarly positive for CD29, CD44, CD49e, and Sca-1, and negative for CD3, CD19, CD31, CD34, CD45, and MHCII) (Supplementary Figure 3).

**FIGURE 1:**
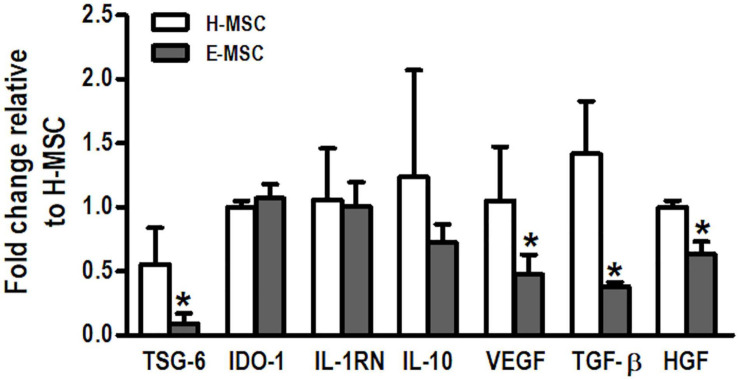
Charaterization of mediators produced by MSCs from healthy and emphysematous donors. H-MSCs, bone marrow-derived mesenchymal stromal cells (BM-MSCs) obtained from healthy donors; E-MSCs, BM-MSCs obtained from emphysematous donors. Gene expression profile of tumor necrosis factor α-induced protein-6 (TSG-6), indoleamine 2,3-dioxygenase 1 (IDO-1), interleukin-1 receptor antagonist (IL-1RN), interleukin 10 (IL-10), vascular endothelial growth factor (VEGF), transforming growth factor β (TGF-β), and hepatocyte growth factor (HGF) in H-MSCs and E-MSCs. Data are expressed as means ± standard deviation of 5–6 samples in each group. *P < 0.05 vs. the H-MSC group.

Compared with H-MSCs, E-MSCs demonstrated lower mRNA expression levels of TSG-6, VEGF, TGF-β, and HGF. No differences were observed in expression levels of IDO-1, IL-1RN, and IL-10 between H-MSCs and E-MSCs (Figure 1).

**FIGURE 2:**
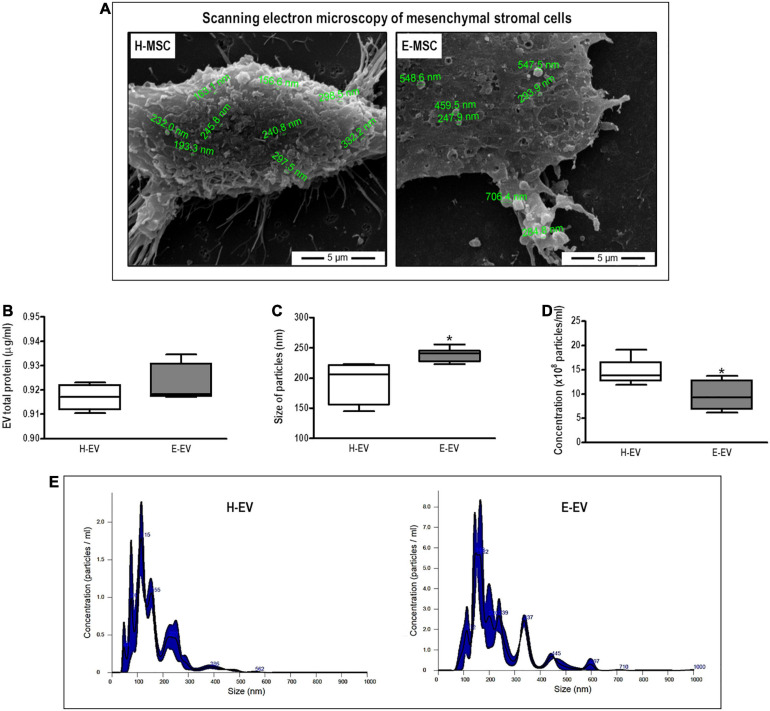
Characterization of EVs secreted by MSCs from healthy and emphysematous donors. H-MSCs, bone marrow-derived mesenchymal stromal cells (BM-MSCs) obtained from healthy donor; E-MSCs, BM-MSCs obtained from emphysematous donors; H-EVs, extracellular vesicles derived from BM-MSCs obtained from healthy donors; E-EVs, extracellular vesicles derived from BM-MSCs obtained from emphysematous donors. **(A)** Representative scanning electron microscopy of H-MSCs and E-MSCs, illustrating EV release from the surface of both types of MSCs. **(B–D)** Characterization of H-EVs and E-EVs, as determined by total protein content **(B)** and the size **(C)**, concentration **(D)**, and distribution **(E)** of secreted particles, measured by the Bradford assay and nanoparticle tracking analysis, respectively. Data are expressed as medians (interquartile range) of 5–6 samples in each group. *P < 0.05 vs. the H-EV group.

H-MSCs and E-MSCs were able to release EVs as confirmed by scanning electron microscopy (Figure 2A), with no difference in total protein content between EVs from both cell types (Figure 2B). However, NTA measurements revealed morphological differences between EVs from H-MSCs and E-MSCs; E-EVs demonstrated larger median diameter and lower average concentration compared with H-EVs (Figures 2C,D), although both H-EVs and E-EVs were also found to be composed of particle populations with more than one peak in the diameter (Figure 2E). H-EVs and E-EVs were isolated, incubated with beads conjugated with anti-CD63 or anti-CD81 antibodies, and analyzed by flow cytometry to demonstrate the presence of vesicles positive for MSC-EV surface markers. Quantitatively, CD63^+^ EVs constituted 8.6 ± 0.03 and 4.5 ± 0.76% of the total H-EVs and E-EVs, respectively, while CD81^+^ EVs constituted 14.9 ± 0.72 and 11.9 ± 0.93% of total H-EVs and E-EVs, respectively (Supplementary Figure 4).

### Characterization of Mitochondrial Function in H-MSCs and E-MSCs

**FIGURE 3:**
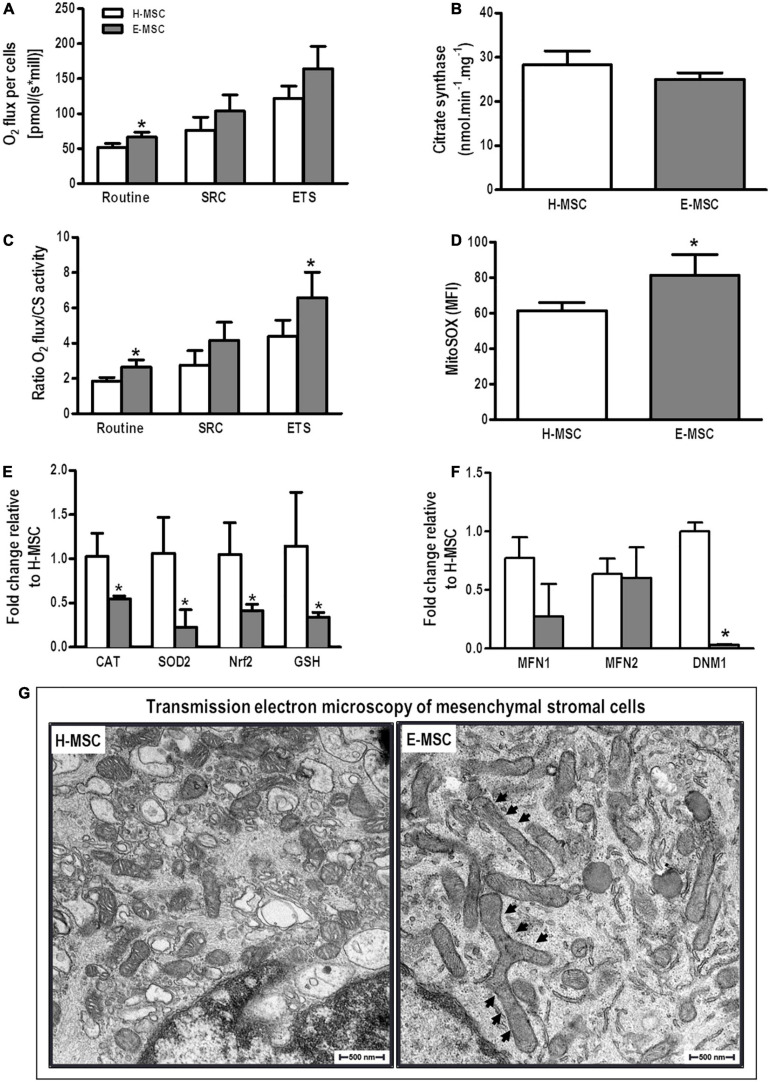
Characterization of mitochondria of MSCs from healthy and emphysematous donors. **(A)** An OROBOROS system was used to measure basal (Routine), spare respiration capacity (SPC) and maximum (electron transfer system, ETS) mitochondrial respiration in H-MSCs and E-MSCs. **(B)** Citrate synthase was used as a quantitative enzyme marker for the presence of intact mitochondria in H-MSCs and E-MSCs. **(C)** Oxygraphy parameters were normalized by citrate synthase activity. **(D)** Mean fluorescence intensity detected by MitoSOX-based flow cytometry in H-MSCs and E-MSCs. Quantitative analysis of several antioxidant **(E)** and mitochondrial dynamics-related **(F)** genes in H-MSCs and E-MSCs by real-time RT-PCR. **(G)** Transmission electron microscopy to assess morphological features of mitochondria in H-MSCs and E-MSCs. Note mitochondrial elongation in E-MSCs (arrows). CAT, catalase; SOD2, superoxide dismutase 2; Nrf2, nuclear factor erythroid 2-related factor; GSH, glutathione; MFN1, mitofusin-1; MFN2, mitofusion-2; DNM1, dynamin-1. Data are expressed as means ± standard deviation of 5–6 samples in each group. *P < 0.05 vs. the H-MSC group.

Mitochondrial respiration was measured in the presence or absence of oligomycin, FCCP, and rotenone/antimycin A to distinguish ATP-linked respiration from proton leak, as described elsewhere (Jackson et al., 2016). E-MSC mitochondria demonstrated higher routine O_2_ consumption than H-MSCs (Figure 3A). After normalization of O_2_ consumption by citrate synthase activity (Figure 3B), E-MSC mitochondria also demonstrated greater maximal O_2_ consumption (electron transfer system capacity) compared with H-MSC mitochondria (Figure 3C). No difference was observed in extracellular hydrogen peroxide (H_2_O_2_) release by mitochondria from both H-MSCs and E-MSCs (data not shown).

Mitochondrial superoxide production is a major cause of the cellular oxidative damage that may underlie degradative diseases and aging. E-MSCs demonstrated significant higher mitochondrial superoxide production compared with H-MSCs (Figure 3D). Furthermore, E-MSCs exhibited a considerable reduction in mRNA expression of catalase (CAT), SOD2, Nrf2, and GSH compared with H-MSCs (Figure 3E).

**FIGURE 4:**
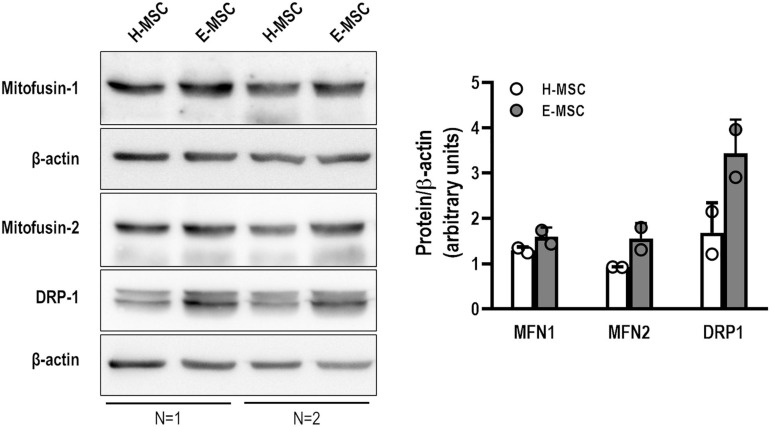
Representative immunoblotting showing mitofusin-1 (MFN1), mitofusin-2 (MNF2), dynamin-related protein 1 (DRP1), and β-actin in MSCs from health (H) and emphysematous (E) donors. Data are expressed (right panel) as the ratio between protein expression and β-actin obtained by densitometry of 2 samples in each group.

To assess putative mitochondrial fusion or fission events, mRNA expression of MFN1, MFN2, and DNM1 (dynamin-related protein-1 encoding gene) was evaluated. There was no difference in expression levels of MFN1 and MFN2, although DNM1 expression was significantly reduced in E-MSCs compared with H-MSCs (Figure 3F). Furthermore, transmission electron microscopy revealed the presence of elongated giant mitochondria in E-MSCs (Figure 3G). To further assess these findings, protein levels of MFN1, MFN2, and DRP-1 (dynamin-related protein-1) were evaluated in both H-MSCs and E-MSCs by immunoblotting. A trend toward increased levels of MFN1, MFN2 and DRP-1 levels was observed in E-MSCs compared to H-MSCs (Figure 4), which corroborates the presence of elongated giant mitochondria in the former cells.

### Evaluation of the Ability of MSCs and Their EVs to Promote Mitochondrial Transfer and AM Modulation *in vitro*

**FIGURE 5:**
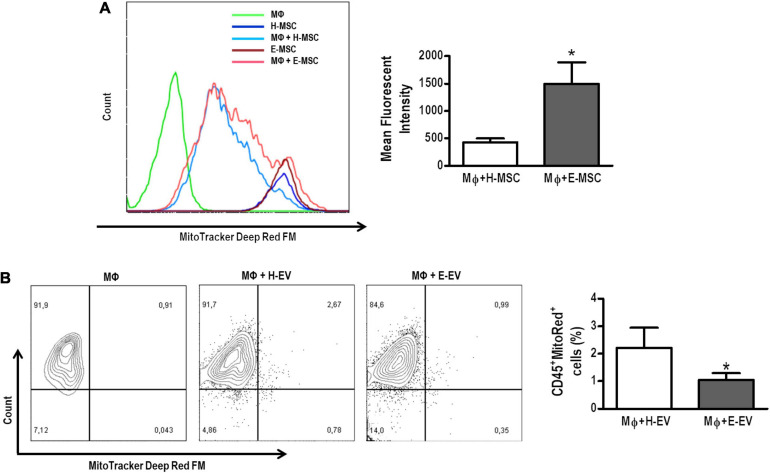
Mitochondrial transfer from healthy or emphysematous MSCs and EVs to alveolar macrophages (MΦ). **(A)** Intensity of MitoRed fluorescence of healthy (H) or emphysematous (E) MSCs decreased after direct co-culture with MΦ (light blue and pink histograms, respectively). The extent of mitochondrial transfer to MΦ was measured by flow cytometry. Co-culture of MΦ with MitoRed-pretreated H-MSCs and E-MSCs resulted in increased MitoRed (APC^+^) mean fluorescent intensity of MΦ. **(B)** Scatter plots show MΦ and EV after 24 h in co-culture. The presence of CD45^+^ MΦ, demonstrating acquisition of MitoRed fluorescence (APC^+^), indicates that mitochondrial transfer occurs from H-EVs and E-EVs. Data are representative of three independent experiments. The histograms represented graphically in **(A)** are from a single representative experiment. *P < 0.05 vs. the MΦ + H-MSC.

AMs co-cultured with E-MSCs demonstrated higher density of MitoTracker deep red (light pink histogram) compared with those co-cultured with H-MSCs (light blue histogram) (Figure 5A). Such findings might be interpreted as a single macrophage tending to receive more mitochondria from E-MSCs (than H-MSCs), or that these cells tend to donate more mitochondria than H-MSCs. Conversely, H-EVs were found to donate more mitochondria to AMs than E-EVs, as assessed by an increase in the percentage of CD45^+^MitoTracker^+^ cells (Figure 5B).

To evaluate whether there was improvement of oxidative capacity of AMs induced by mitochondrial transfer, O_2_ consumption by AMs was measured after 24 h of direct co-culture of these cells with H-EVs or E-EVs (Supplementary Figure 5). Although there was no difference between groups in the routine O_2_ consumption, AMs exhibited significant increases in spare respiratory capacity and in maximal O_2_ consumption after 24 h of co-culture with H-EVs (Supplementary Figure 5).

**FIGURE 6:**
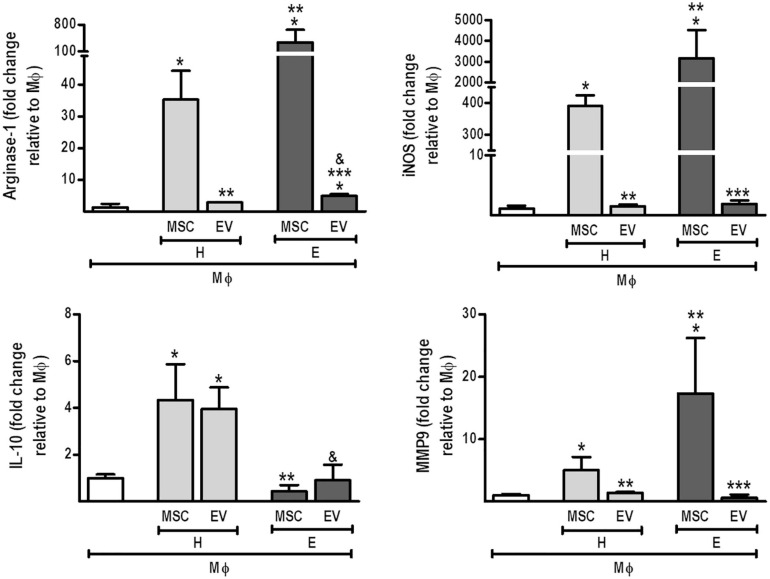
Direct co-culture of MSCs and EVs from healthy or emphysematous donors modulates the alveolar macrophage (MΦ) profile. H-MSCs, 1 × 10^6^ bone marrow-derived mesenchymal stromal cells (BM-MSCs) obtained from healthy donors; E-MSCs, 1 × 10^6^ BM-MSCs obtained from emphysematous donors; H-EVs, extracellular vesicles derived from 1 × 10^6^ BM-MSCs obtained from healthy donors; E-EVs, extracellular vesicles derived from 1 × 10^6^ BM-MSCs obtained from emphysematous donors. Data are expressed as medians (interquartile range) of 5–6 samples in each group. *P < 0.05 vs. MΦ. **P < 0.05 vs. the H-MSC. ***P < 0.05 vs. the E-MSC. ^&^*P* < 0.05 vs. the H-EV.

AMs co-cultured with H-MSCs demonstrated an increase in mRNA levels of arginase-1 and IL-10 (Figure 6). H-MSCs also promoted an increase in expression levels of iNOS and MMP9, although these were even greater when AMs were co-cultured with E-MSCs. Furthermore, AMs co-cultured with E-MSCs demonstrated increased mRNA levels of arginase-1. H-EVs, but not E-EVs, were able to induce an increase in IL-10 expression by AMs.

### Effects of MSCs and EVs From Healthy and Emphysematous Donors in a Murine Model of Severe Emphysema

**FIGURE 7:**
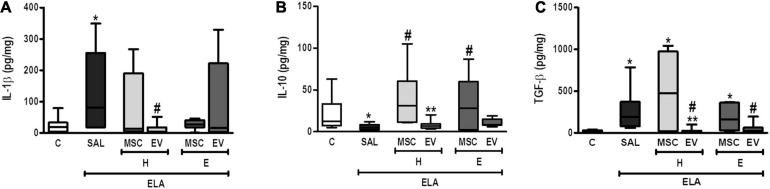
Levels of IL-1β **(A)**, IL-10 **(B)**, and TGF-β **(C)** in lung tissue. C, intratracheal instillation of 50 μL of saline; ELA, intratracheal instillation of 0.2 IU of pancreatic porcine elastase; SAL, intravenous injection of 50 μL of saline; H-MSCs, 1 × 10^6^ bone marrow-derived mesenchymal stromal cells (BM-MSCs) obtained from healthy donors; E-MSCs, 1 × 10^6^ BM-MSCs obtained from emphysematous donors; H-EVs, extracellular vesicles derived from 1 × 10^6^ BM-MSCs obtained from healthy donors; E-EVs, extracellular vesicles derived from 1 × 10^6^ BM-MSCs obtained from emphysematous donors. IL-1β, interleukin-1β; IL-10, interleukin-10; TGF-β, transforming growth factor-β. Data are expressed as means ± standard deviation of 7–10 animals/group. *P < 0.05 vs. the C group. ^#^*P* < 0.05 vs. the ELA-SAL group. **P < 0.05 vs. the H-MSC group.

Compared with the C group, ELA-SAL animals demonstrated increased protein levels of IL-1β and TGF-β and reduced IL-10 levels in lung tissue (Figure 7). Both H-MSCs and E-MSCs led to an increase in IL-10 levels, but had no significant effects on IL-1β and TGF-β levels in comparison with ELA-SAL. On the other hand, H-EVs and E-EVs were able to reduce TGF-β levels. Furthermore, H-EVs, but not E-EVs, reduced IL-1β levels compared with ELA-SAL animals.

E-SAL animals demonstrated a reduction in static lung elastance, as well as increased Lm and neutrophils in lung tissue (Table 1). Neither MSCs nor EVs from healthy and emphysematous donors improved static lung elastance in the emphysema mice. Only H-EVs were able to significantly reduce Lm and the neutrophil cell count in lung tissue in comparison with ELA-SAL animals.

**TABLE 1:** Lung mechanics and morphometry.

		Est,L (cmH_2_O)	Lm (μ m)	Neutrophils (%)
**C**		30.8 ± 2.2	25.4(23.4−26.9)	1.2 ± 0.9
**ELA**				
SAL		21.6 ± 3.3*	51.5(39.5−138.7)*	10.7 ± 5.3*
H	MSC	22.7 ± 8.1	84.1(41.7−113.1)	7.1 ± 4.0*
	EV	24.4 ± 3.0	27.2(26.4−39.9)^#^	2.9 ± 0.9^#^
E	MSC	26.4 ± 7.0	36.7(28.7−82.2)	6.2 ± 2.0*
	EV	22.3 ± 4.8	92.8(28.6−102.3)	8.2 ± 2.2^*,&^

Values are means ± standard deviation, for parametric data, or median (interquartile range), for non-parametric data, of 7–10 mice in each group. All values were computed in 10 random, non-coincident fields per animal. In the control (C) group, saline was instilled intratracheally. In the emphysema (ELA) groups, mice received porcine pancreatic elastase intratracheally. At day 21, all groups were randomized to receive saline (SAL), bone marrow-derived mesenchymal stem cells (MSCs) from healthy (H) or emphysematous (E) donors (1 × 10^6^ cells), or BM-MSC-derived extracellular vesicles (EVs) from healthy or emphysematous donors (proportional volume obtained from 10^6^ BM-MSCs), intratracheally. Est, L, static lung elastance; Lm, mean linear intercept. *P < 0.05 vs. the C group. ^#^P < 0.05 vs. the ELA-SAL group. ^&^P < 0.05 vs. the H-EV group.

**FIGURE 8:**
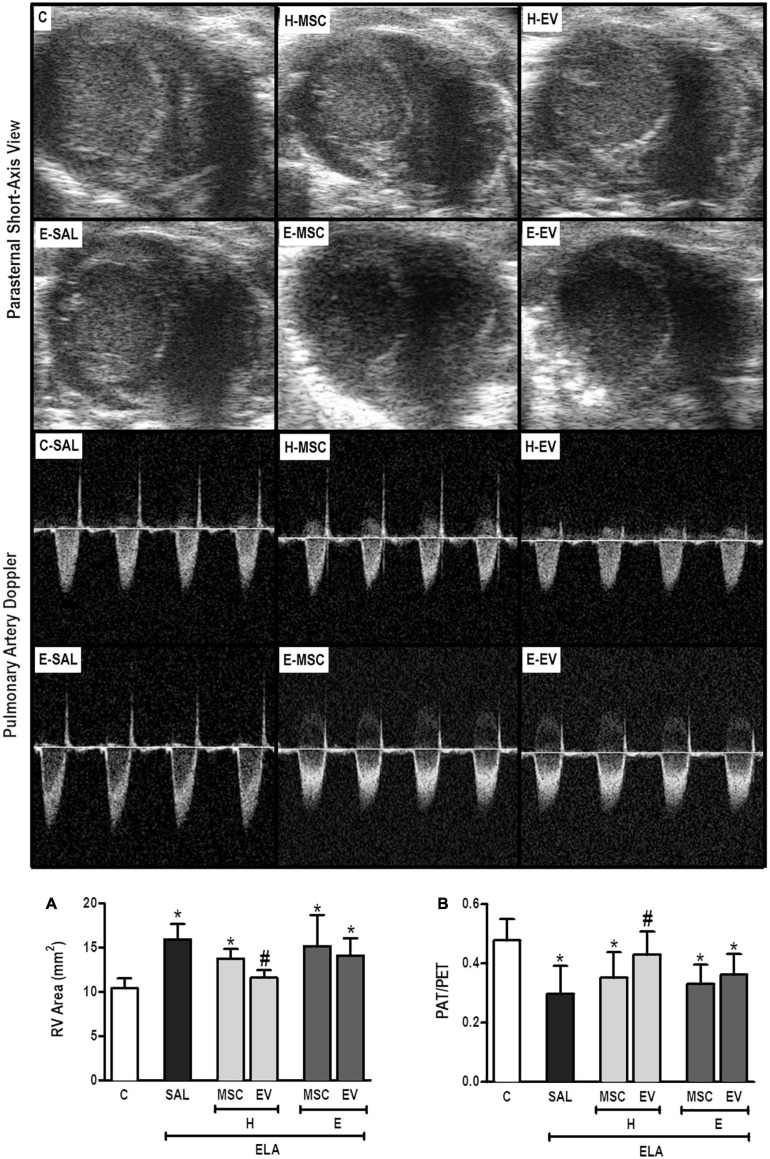
Echocardiographic analysis. Parasternal short axis view of both ventricles (upper panel) and pulmonary artery flow dynamics (lower panel). **(A)** Pulmonary artery acceleration time/pulmonary artery ejection time ratio (PAT/PET) and **(B)** right ventricle area. C, intratracheal instillation of 50 μL of saline; ELA, intratracheal instillation of 0.2 IU of pancreatic porcine elastase; SAL, intravenous injection of 50 μL of saline; H-MSCs, 1 × 10^6^ bone marrow-derived mesenchymal stromal cells (BM-MSCs) obtained from healthy donors; E-MSCs, 1 × 10^6^ BM-MSCs obtained from emphysematous donors; H-EVs: extracellular vesicles derived from 1 × 10^6^ BM-MSCs obtained from healthy donors; E-EVs, extracellular vesicles derived from 1 × 10^6^ BM-MSCs obtained from emphysematous donors. RV, right ventricle. Data are expressed as means ± standard deviation of 7–10 animals/group. *P < 0.05 vs. the C group. ^#^*P* < 0.05 vs. the ELA-SAL group.

E-SAL animals demonstrated the characteristics of cardiovascular dysfunction secondary to emphysema (*cor pulmonale*), which include pulmonary artery hypertension (reduced PAT/PET ratio) and increased right ventricular area (Figure 8). Only H-EVs were able to significantly increase the PAT/PET ratio and reduce the right ventricular area to values comparable with controls.

**FIGURE 9:**
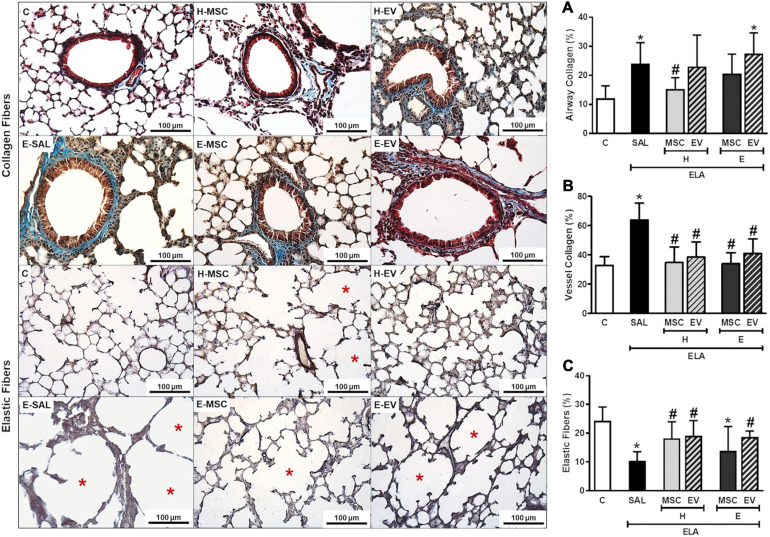
Effects of H-MSCs, E-MSCs, H-EVs or E-EVs treatments on the remodeling process. Left panels: representative photomicrographs of lung parenchyma stained with Masson’s trichome staining collagen fibers in airways, **(A)**; walls of blood vessels in interlobular septa, **(B)** and Weigert’s resorcin fuchsin elastic fibers in alveolar septa, **(C)**. Note that blood vessels are highlighted by red arrowheads, elastic fibers by black arrowheads, and hyperinflated areas by red asterisks. C: intratracheal instillation of 50 μL of saline; ELA: intratracheal instillation of 0.2 UI of pancreatic porcine elastase (PPE); SAL: intravenous injection of 50 μL of saline; H-MSCs: 1 × 10^6^ bone marrow mesenchymal stromal cells obtained from healthy donor; E-MSCs: 1 × 10^6^ bone marrow mesenchymal stromal cells obtained from emphysematous donor; H-EVs: extracellular vesicles derived from 1 × 10^6^ bone marrow mesenchymal stromal cells obtained from healthy donor; E-EVs: extracellular vesicles derived from 1 × 10^6^ bone marrow mesenchymal stromal cells obtained from emphysematous donor. Right panels: Data are expressed as mean + SD of 5–7 animals/group. *vs. C group (*p* < 0.05). ^#^Vs. ELA-SAL group (*p* < 0.05).

E-SAL animals demonstrated increased collagen deposition in airways, interlobular septa, and walls of blood vessels, as well as a reduction in elastic fiber content in the alveolar septa. All therapies administered were able to induce significant reductions in collagen deposition in walls of blood vessels in interlobular septa (Figure 9). Only H-MSCs reduced airway collagen content, while percentage of elastic fiber in alveolar septa was increased after H-MSC, H-EV, and E-EV therapies.

## Discussion

To the best of our knowledge, this is the first study to comparatively evaluate the therapeutic potential of MSCs and EVs obtained from healthy and emphysematous donors. *In vitro* characterization of H-MSCs and E-MSCs demonstrated that the latter exhibited reduced mRNA expression of anti-inflammatory, antioxidant, and immunomodulatory mediators as well as dysfunctional mitochondria. By using a multiple-dose protocol of elastase-induced emphysema that resembles advanced human emphysema, which has been extensively characterized in our laboratory (Antunes et al., 2014; Padilha et al., 2015; Henriques et al., 2016; Oliveira et al., 2016; Poggio et al., 2018), we demonstrated that E-MSCs and E-EVs did not yield the same therapeutic benefits as those of H-MSCs and H-EVs. In fact, E-MSCs and their EVs did not exert any beneficial effect on lung mechanics, histology, or cardiac function *in vivo.* On the other hand, H-EVs appeared to be the best candidate for the treatment of severe emphysema, because these derivates were able to concomitantly reduce Lm and neutrophil infiltration in lung tissue as well as cardiovascular dysfunction.

The beneficial effects of MSCs in experimental studies encouraged several clinical trials on individuals with COPD. A good safety profile was demonstrated for administration of allogeneic MSCs (Weiss et al., 2013; Oliveira et al., 2017), although efficacy remains to be demonstrated in future clinical studies. Low viability and decreased functionality of MSCs soon after transplantation might account for a reduced efficacy (Li and Lin, 2012; Li et al., 2016). An indispensable requirement in allogeneic transplantation is low to negligible expression of MHC molecules. Recent evidence demonstrated that MHC molecules are minimally expressed on unpassaged MSCs but are progressively upregulated when cells are expanded in culture. Furthermore, the surface molecule, CD59, which prevents complement attack, is downregulated with passaging (Moravcikova et al., 2018). A recent report demonstrated that HLAs on adipose tissue-derived MSCs are the major cause of alloreactive CD8^+^ T cell-mediated cytotoxicity through granzyme B secretion; HLA antibody blockade promptly reduced this in MSCs (Chang and Park, 2019). In this context, a previous study observed that autologous MSCs are less susceptible to cellular injury after serum contact compared with allografts (Li and Lin, 2012). Such findings suggest that autologous transplantation might be considered a better option for cell therapy because it might allow an increase in viable MSC counts after transplant.

No obvious differences in the structural characteristics of bone marrow have been reported between individuals with or without COPD (Broekman et al., 2016; Toledo-Pons et al., 2018), except for a reduction in the number of circulating hematopoietic and endothelial progenitor cells in individuals with COPD (Huertas et al., 2010; Janssen et al., 2014). In a prospective clinical trial, BM-MSCs were autologously administered in individuals with severe emphysema as an adjunct therapy to lung volume reduction surgery (Stolk et al., 2016). Although autologous MSC therapy was demonstrated to be feasible and safe, no further therapeutic effects were observed by MSC administration compared with lung volume reduction surgery alone (Stolk et al., 2016). In the current study, we found that TSG-6, VEGF, TGF-β, and HGF are downregulated in E-MSCs, which may reduce the therapeutic effects, because these mediators are associated with anti-inflammatory and reparative actions. In this context, TSG-6 was reported to inhibit neutrophil chemotaxis mediated by CXCL8 (Dyer et al., 2014; Abreu et al., 2019). Furthermore, MSCs have been shown to inhibit proinflammatory response, cell apoptosis, and excessive protease expression by upregulating VEGF, TGF-β, and HGF in experimental emphysema (Guan et al., 2013; Kennelly et al., 2016).

EV secretion has been considered a major mechanism by which MSCs promote therapeutic actions. These small extracellular membrane fragments are heterogeneous in origin, dimension, and content (Abreu et al., 2021). Previous studies revealed that a same type of cell may release different subtypes of EVs, as uncovered by distinct small-RNA enrichment signatures (Ji et al., 2014; Than et al., 2020). In another study, three different subpopulations of EVs enriched in exosomal markers and miRNAs were also isolated from MSCs by gradient flotation (Collino et al., 2017), and only the EV fraction with medium flotation density was able to stimulate cell proliferation and inhibit renal tubular cell apoptosis in a model of ischemia-reperfusion injury (Collino et al., 2017). In the current study, we used the entire fraction of EVs obtained by ultracentrifugation, without sorting any subpopulation out by gradient processes. NTA measurement revealed that more than one diameter peak was found within each EV population; however, E-MSCs produced a range of particles with a larger median diameter, and a higher concentration of EVs was obtained from H-MSCs. Such findings might account, at least partially, for the distinct therapeutic actions exerted by these MSCs and their derivates.

Under normal physiological conditions, redox homeostasis is maintained by a balance between mitochondria fission and fusion events as well as production of ROS and antioxidants. While mitochondrial fusion is stimulated by energy expenditure and cellular stresses, mitochondrial fission is associated with mitochondrial quality control (Youle and van der Bliek, 2012; Antunes et al., 2021). However, persistent exposure to noxious particles or gases may lead to abnormalities in mitochondrial morphology and function, thus resulting in excessive production of ROS and tissue damage (Antunes et al., 2021). In mouse embryonic fibroblasts, a reduction in mitochondrial fusion was associated with decreased mitochondrial oxygen consumption and membrane potential (Chen et al., 2005), and elongated giant mitochondria induced by the blockade of mitochondrial fission in hepatocytes were sufficient to develop senescent phenotypes with increased production of ROS (Yoon et al., 2006). In the current study, we found that E-MSCs present alterations in both mitochondrial function and antioxidant capacity with considerably reduction in expression levels of CAT, SOD, Nrf2, and GSH. E-MSCs also demonstrated a trend to increase protein expression of MFN1 and MFN2, which are involved in mitochondrial fusion. In additional, levels of DNM-1 (a mitochondrial fission-related gene) were downregulated in E-MSCs, while a trend toward increased protein levels of dynamin-related protein 1 (DRP-1) was also observed, probably as a compesatory mechanism. These findings are indeed consistent with the presence of elongated giant mitochondria in E-MSCs. Similar observations were found in senescent cancer cells with mitochondrial dysfunction (Martínez et al., 2019). In addition to protein expression, mitochondrial fusion/fission balance is highly regulated by phosphorylation. Accordingly, post-translational modifications in MFN1 (Pyakurel et al., 2015), MFN2 (Leboucher et al., 2012), and DRP-1 (Taguchi et al., 2007) (e.g., phosphorylation) have been implicated in their activity and in mitochondrial dynamics. However, in the present study, only the total protein levels of MFN1, MFN2, and DRP-1 were assessed.

In previous studies using experimental models of acute respiratory distress syndrome, mitochondrial transfer from MSCs to host cells was associated with enhanced macrophage phagocytic activity and antimicrobial effects (Jackson et al., 2016; Morrison et al., 2017) as well as recovered alveolar ATP concentration (Islam et al., 2012). Systemic administration of a mitochondria-rich fraction isolated from MSCs was also effective at reducing lung, kidney and liver damage in experimental sepsis (de Carvalho et al., 2021). Although both H-MSCs and E-MSCs used here indicated transfer of their mitochondria when co-cultured with AMs, E-MSC mitochondria might be less effective at reducing harmful effects from noxious environment because these demonstrated a senescent phenotype. AMs co-cultured with E-MSCs demonstrated a much higher expression of iNOS, which may be correlated to the downregulation of antioxidants and increases in the cellular oxygen consumption rate and superoxide production induced by E-MSC mitochondria. AMs co-cultured with either H-MSCs or E-MSCs also demonstrated upregulation of arginase-1. These phagocytes use the mutually substrate-competitive enzymes iNOS and arginase-1 to metabolize L-arginine, thus driving cellular polarization to an M1 pro-inflammatory or an M2 anti-inflammatory phenotype (El-Gayar et al., 2003; Eapen et al., 2017). Nevertheless, only AMs co-cultured with H-MSCs and H-EVs demonstrated upregulation of IL-10, which exerts anti-inflammatory and reparative actions.

Both chronic inflammation and oxidative damage cause progressive alterations in lung structure. An intense neutrophil influx occurs during the development of emphysema, and these cells cause elastolysis and enlargement of airspaces by secreting multiple proteases, including neutrophil elastase. In addition to the detrimental effects on lung function, the disease also leads to cardiovascular impairment (Barnes et al., 2015). In our *in vivo* model of emphysema, multiple instillations of elastase resulted in an increase in neutrophil count, Lm, elastolysis, and peribronchiolar and perivascular fibrosis, as well as impaired cardiorespiratory function. Although both H-MSCs and E-MSCs increased pulmonary IL-10 levels and reduced collagen content in lung tissue, they did not reverse lung hyperinflation and cardiopulmonary dysfunction in experimental severe emphysema, which corroborates with data from early stage clinical trials that demonstrated no efficacy of allogeneic or autologous MSC administration in individuals with COPD (Weiss et al., 2013; Stolk et al., 2016). However, this is in contrast to our previous investigations that demonstrated therapeutic actions induced by H-MSCs (Antunes et al., 2014; Poggio et al., 2018). Some potential explanations for these results include differences in experimental design: route of MSC administration (intratracheal vs. intravenous), therapeutic window (3 h vs. 24 h after last instillation), and emphysema severity (0.1 vs. 0.2 IU PPE per instillation, i.e., moderate vs. severe disease)—which may account for a potential low viability and decreased functionality of MSCs soon after transplantation. Such findings demonstrate an important role of these factors from a translational perspective. Alternatively, EVs retain several characteristics of their originating cells and can interpose paracrine effects and offer several advantages as a cell-free therapy (Abreu et al., 2021): (1) lower risk of triggering innate and adaptive immune responses; (2) high stability; (3) suitability for long-term storage with no cryoprotective agents; (4) ability to travel systemically without the risk of clumping; (5) migration to target tissues where MSCs can cross; and (6) suitability for mass production. E-EVs reduced TGF-β levels in lung tissue but were unable to mitigate cardiorespiratory dysfunction in emphysematous mice. On the other hand, H-EVs significantly reduced lung inflammation and damage, and improved cardiovascular function with reversal of pulmonary hypertension (PAT/PET) and right ventricular dysfunction, demonstrating the potential of these H-MSCs derivates to promote therapeutic actions *in vivo*.

Some limitations of our study should be considered: (1) the absence of characterization of H-EV and E-EV content (i.e., by proteomic analysis), which limited clarification of why H-EVs were therapeutically better than E-EVs; (2) use of the total content of MSC-derived EVs, with no isolation of the different subpopulations (i.e., exosomes, microvesicles, apoptotic bodies) contained in each type of EV, which could have helped explain whether a specific subpopulation played a preponderant role in the beneficial effects of H-EVs; (3) the EV dose was proportional to the amount of vesicles obtained from 1,000,000 MSCs (H or E). However, E-MSCs were shown to release fewer EVs than H-MSCs under the same conditions, which could add bias to our study, because E-EVs were therapeutically inefficient compared with H-EVs.

## Conclusion

Our results demonstrated that H-EVs were the best therapeutic tool in the model of elastase-induced severe emphysema, which was characterized by significant vascular bed impairment. On the other hand, E-MSCs and their EVs were unable to positively alter any of the experimental endpoints. This lack of effect was followed by increased ROS production, reduced antioxidant and immunomodulatory capacities, and the presence of abnormal mitochondria in E-MSCs. We believe that the better performance of H-EVs in comparison with H-MSCs is due to several advantages of these small packages, which retain many characteristics of their originating cells and easily get to more distal and branched areas that MSCs are unable to reach. Finally, our results suggest that novel strategies to potentiate the bioenergetics of MSCs obtained from patients with severe COPD could enable future clinical trials using autologous MSCs (or their products) in these patients.

## Data Availability Statement

The original contributions presented in the study are included in the article/Supplementary Material, further inquiries can be directed to the corresponding author/s.

## Ethics Statement

This study was approved by the Ethics Committee of the Federal University of Rio de Janeiro Health Sciences Center (CEUA-UFRJ: 014/14, Rio de Janeiro, Brazil). All animals received humane care by trained veterinarians and veterinary staff in compliance with the “Principles of Laboratory Animal Care” formulated by the National Society for Medical Research and the U.S. National Academy of Sciences Guide for the Care and Use of Laboratory Animals.

## Author Contributions

MA, DW, JL, ML-P, FC, and PR conceived and designed the study. MA, CB, TO, JK, LC, DX, MC, NR, RS-A, CC-N, and CC performed the experiments. MA, CB, JK, EM, AG, RS-A, CC-N, JL, ML-P, FC, and PR analyzed and interpreted the data. DW and JL review for intellectual content. MA, ML-P, FC, and PR wrote the manuscript. All authors read and approved the final version of the manuscript.

## Conflict of Interest

The authors declare that the research was conducted in the absence of any commercial or financial relationships that could be construed as a potential conflict of interest.
