# Supplementary material for: Mesenchymal Stromal Cells From Emphysematous Donors and Their Extracellular Vesicles Are Unable to Reverse Cardiorespiratory Dysfunction in Experimental Severe Emphysema
Source: Front Cell Dev Biol. 2021 May 31;9:661385. doi: 10.3389/fcell.2021.661385 (PMC8202416; doi:10.3389/fcell.2021.661385)
Supplement: Supplementary file 1 [file Data_Sheet_1.PDF]

Supplementary Material

1.1 Supplementary Figures

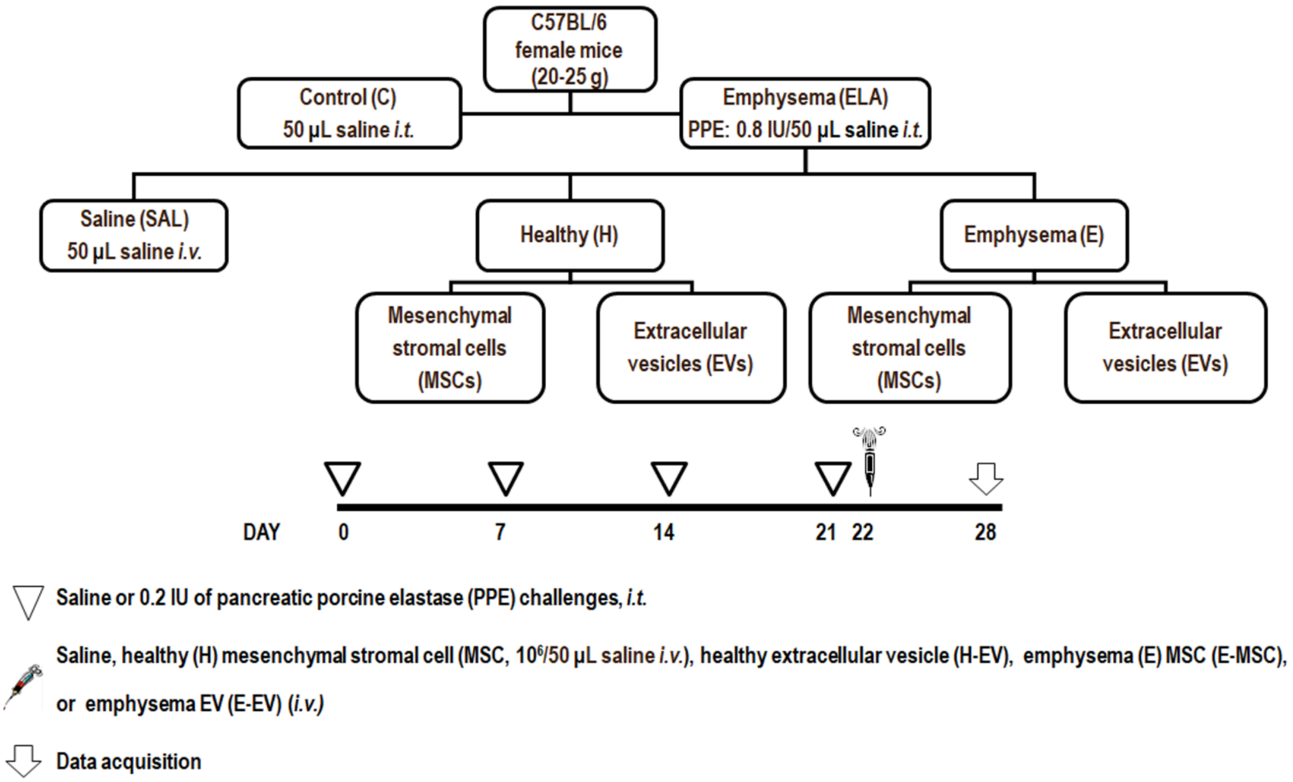

Supplementary Figure 1. Schematic flow chart and timeline of study design. C: instillation of 50 µL of saline; ELA: instillation of 0.8 IU of pancreatic porcine elastase (PPE); SAL: intravenous injection of 50 µL of saline; H-MSCs: 1×10^6 bone marrow-derived mesenchymal stromal cells (BM-MSCs) obtained from healthy donor; E-MSCs: 1×10^6 BM-MSCs obtained from emphysematous donor; H-EVs: extracellular vesicles derived from 1×10^6 BM-MSCs obtained from healthy donor; E-EVs: extracellular vesicles derived from 1×10^6 BM-MSCs obtained from emphysematous donor; IV: intravenous injection; IT: intratracheal injection. All data were acquired on day 28.

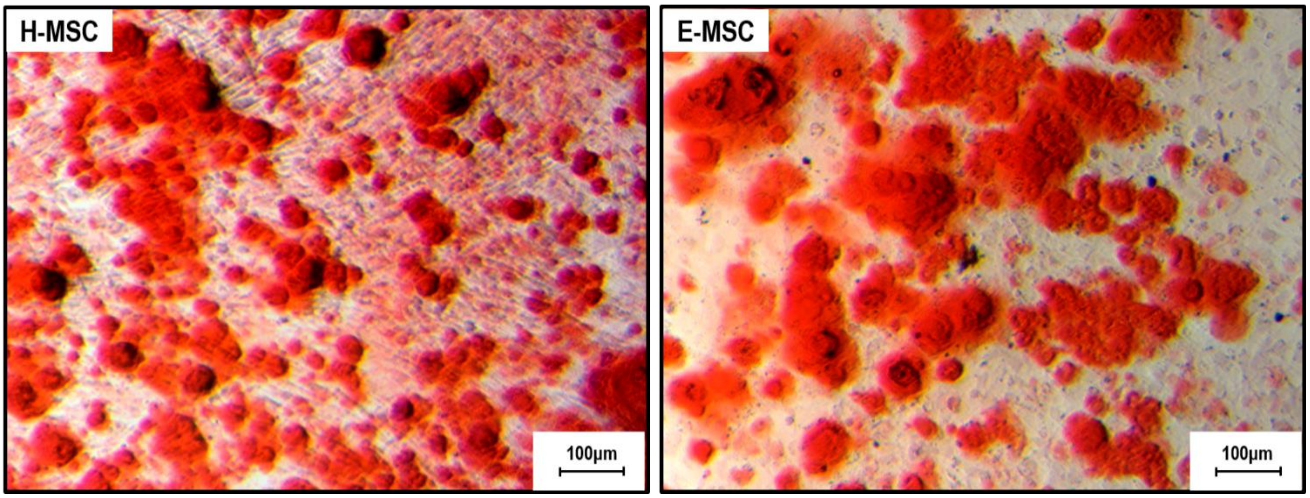

Supplementary Figure 2. Phenotypic characterization of MSCs. Osteogenic (Alizarin Red S staining) differentiation of mesenchymal stromal cells (MSCs) isolated from femur and tibia bone marrow of 8-week-old C57BL/6 mice, either healthy (H-MSC) or emphysematous (E-MSC; previously subjected to our 4-week elastase emphysema protocol). Scale bar: 100µm.

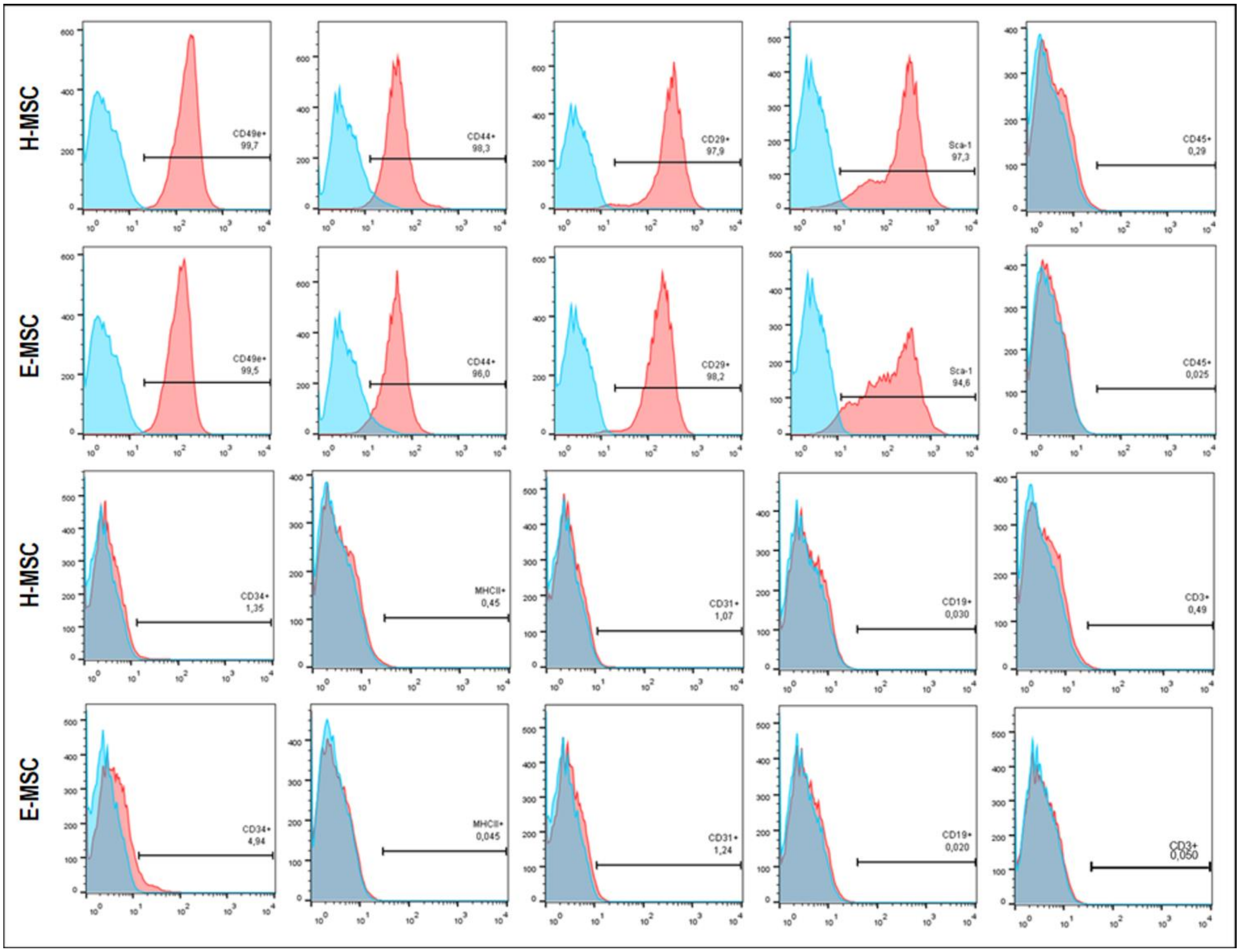

Supplementary Figure 3. Cell characterization by flow cytometry. H-MSCs: bone marrow-derived mesenchymal stromal cells (BM-MSCs) obtained from healthy donor; E-MSCs: BM-MSCs obtained from emphysematous donor. Flow cytometry reveals that mesenchymal stem cells are negative (−) for leukocyte (CD3, CD45), hematopoietic-cell (CD34), antigen-presenting-cell (MHC II), endothelial-cell (CD31), and B-cell (CD19), markers, while they are concomitantly positive (+) for stem-cell (Sca-1) and mesenchymal-cell (CD49e, CD44, CD29) markers.

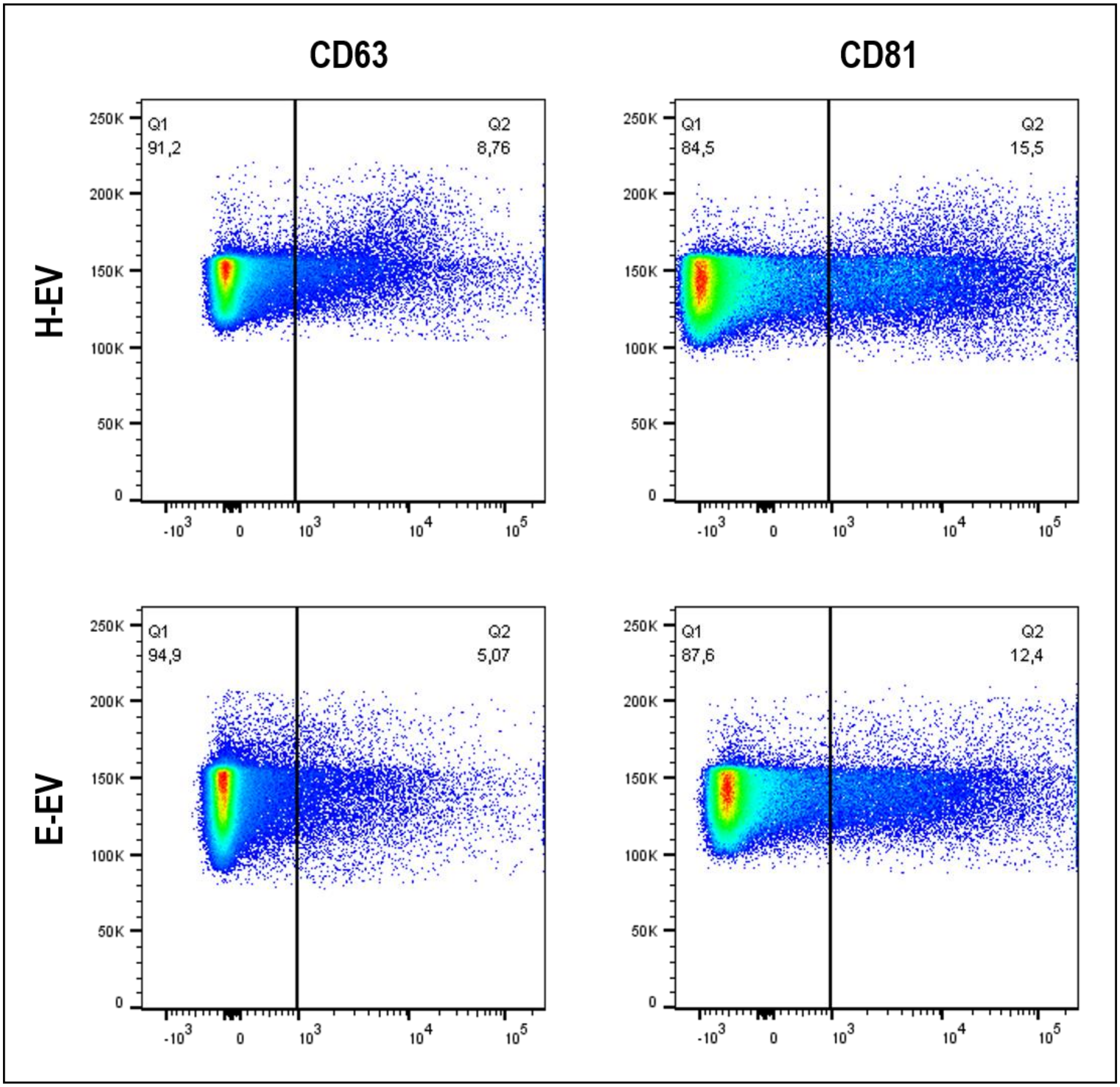

Supplementary Figure 4. Characterization of bone marrow MSC-derived extracellular vesicles (EVs). Representative flow cytometry plots of both healthy (H) and emphysema (E) EVs conjugated with 4-µm beads demonstrating presence of EV populations positive for CD63 (H-EVs: 8.76%; E-EVs: 5.07%) and for CD81 (H-EVs: 15.5%; E-EVs: 12.4%).

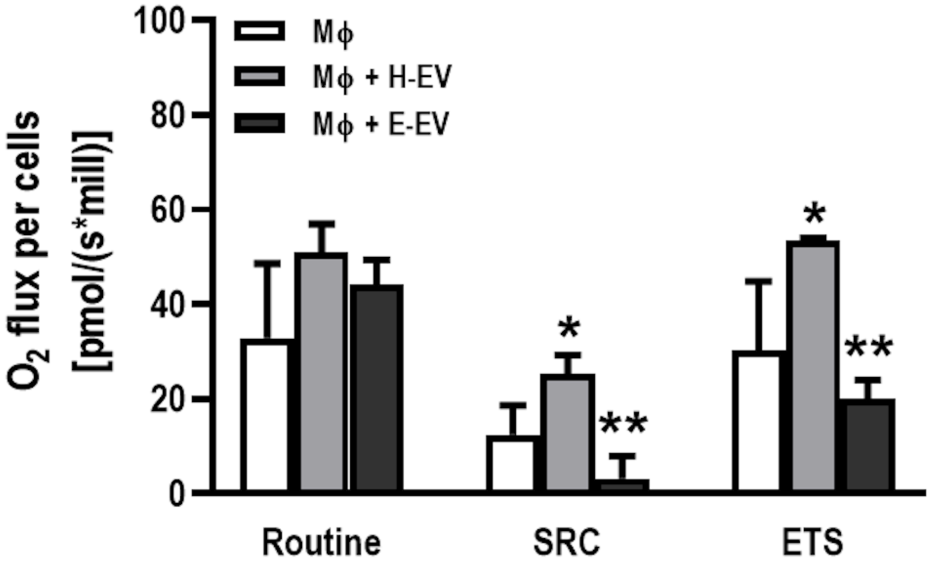

Supplementary Figure 5. Characterization of mitochondria of alveolar macrophages (MΦ) after mitochondrial transfer from healthy (H) or emphysematous (E) EVs. OROBOROS system was used to measure basal (Routine), spare respiration capacity (SPC), and maximum (electron transfer system, ETS) mitochondrial respiration in MΦ after 24h of co-culture with H-EVs and E-EVs. Data are expressed as means ± standard deviation of 3 samples in each group. **P* < 0.05 versus MΦ. ***P* < 0.05 versus MΦ + H-EV.

1.2 Supplementary Table

TABLE S1. Forward and Reverse Oligonucleotide Sequences of Target Gene Primers

Primer ID	Forward Sequence	Reverse Sequence
**DNM1**	5′-ATCTGCAGGTGGTGGGATTG-3′	5′-CTACTCAGGTAGGCAGCAGC-3′
**MFN1**	5′-CTTGCTGTCATTGGGGAGGT-3′	5′-CATCCTCCATGTACTCCGGC-3′
**MFN2**	5′-TCGGAGCCTGAGTACATGGA-3′	5′-GAGAGGCGCCTGATCTCTTC-3′
**TSG6**	5′-ATTTGAAGGTGGTCGTCTCG-3′	5′-TGCATGTGGGTTGTAGCAAT-3′
**IDO1**	5′-ACGGACTGAGAGGACACAGG-3′	5′-CCACCAATAGAGAGACGAGGA-3′
**HGF**	5′-AGCACCATCAAGGCAAGG-3′	5′-GACCAGGAACAATGACACCA-3′
**IL-10**	5′-TCCCTGGGTGAGAAGCTG-3′	5′-GCTCCACTGCCTTGCTCT-3′
**IL-1RN**	5′-AACCACCAGGGCATCACATA-3′	5′-CCTCTTGCCGACATGGAATA-3′
**CAT**	5′-CCTCGTTCAGGATGTGGTTT-3′	5′-TCTGGTGATATCGTGGGTGA-3′
**GSH**	5′-TAATGCGGTGGTGCTACTGA-3′	5′-GCAACTTCCTGGTCATCCAT-3′
**NRF2**	5′-AGCCTCTGTCACCAGCTCA-3′	5′-ATGGGGCTTTTTGATGACC-3′
**SOD2**	5′-CCAAAGGAGAGTTGCTGGAG-3′	5′-GAACCTTGGACTCCCACAGA-3′
**iNOS**	5′-TGGTGGTGACAAGCACATTT-3′	5′-AAGGCCAAACACAGCATACC-3′
**ARG1**	5′-GCTCAGGTGAATCGGCCTTTT-3′	5′-TGGCTTGCGAGACGTAGAC-3′
**TGFβ**	5′-ATACGCCTGAGTGGCTGTC-3′	5′-GCCCTGTATTCCGTCTCCT-3′
**VEGF**	5′-GACCCTGGCTTTACTGCTG-3′	5′-GCTTCGCTGGTAGACATCC-3′
**MMP9**	5′-AGTCCGGCAGACAATCCTT-3′	5′-CCCTGTAATGGGCTTCCTC-3′
**36B4**	5′-CAACCCAGCTCTGGAGAAAC-3′	5′-GTTCTGAGCTGGCACAGTGA-3′

DNM1, dynamin-related protein; MFN1, mitofusin 1; MFN2, mitofusin 2; TSG6, tumor necrosis factor-inducible gene 6 protein; IDO1, indoleamine 2,3-dioxygenase 1; HGF, hepatocyte growth factor; IL-10, interleukin 10; IL-1RN, interleukin 1 receptor antagonist; CAT, catalase; GSH, glutathione; NRF2, nuclear factor erythroid 2-related factor; SOD2, superoxide dismutase 2; iNOS, inducible nitric oxide synthase; ARG1, arginase-1; TGFβ, transforming growth factor; VEGF, vascular endothelial growth factor; MMP9, matrix metalloprotease-9; 36B4, ribosomal protein lateral stalk subunit P0 (housekeeping gene).
